# Supplementary material for: Moxibustion for declined cardiorespiratory fitness of apparently healthy older adults: A study protocol for a randomized controlled trial
Source: PLoS One. 2024 Apr 9;19(4):e0301673. doi: 10.1371/journal.pone.0301673 (PMC11003611; doi:10.1371/journal.pone.0301673)
Supplement: S2 File — (PDF) [file pone.0301673.s002.pdf]

Approval Form for Research Ethical Review in The Second Affiliated Hospital of Nanjing University of Chinese Medicine

Approval No	2023SEZ-007-01		
Project name	Clinical protocol quantification and optimization study of moxibustion to improve cardiorespiratory endurance in the elderly		
Project source	Jiangsu Province Traditional Chinese Medicine Science and Technology Development Plan		
Research Institution	The Second Affiliated Hospital of Nanjing University of Chinese Medicine		
Investigator	Zhang Jianbin		
Approval category	Initial review	Approval procedure	Meeting Review
Review Date	March 2nd,2023	Review site	Jiangsu Second Chinese Medicine Hospital
Juror	Zheng Liang Wang Xia		
Approval files	Assignment for technical design(Version1.0;Date:2023/02/16)<br>Informed consent form(Version1.0;Date:2023/02/16)<br>Case report form(Version1.0;Date:2023/02/16)<br>Treatment diary card		
Review recommendation	According to World Medical Association Declaration of Helsinki Ethical Principles for Medical Research Involving Human Subject，International Ethical Guidelines for Biomedical Research Involving Human Subjects by the Council for International Organization of Medical Science and several Chinese ethical guidelines, assignment for technical design, informed consent form and case report form were approved by the Research Ethical Committee of The Second Affiliated Hospital of Nanjing University of Chinese Medicine.<br>Please carry out the research according to the approval assignment.<br>The investigator should report to the Ethical Committee if the following occurs:<br>1)Amendment of research protocol and informed consent form.<br>2)Change of major investigator.<br>3)Serious adverse events.<br>4)Occurrence of significant influence to the research or increasing harm to participants.<br>5)Deviation of research protocol.		

	6)Research suspension or termination.<br>The research ethical committee will track and check the research process according the specified year or frequency, and the research progress report should be submitted a month before the deadline.<br>Please submit research conclusion report to the research ethical committee.<br>If the project cannot start within the effective period, this document will be invalid and need resubmitting related documents.
frequency of continuing review	12,months<br>Please submit research progress report before March 2nd,2024
Term of validity	2023.03.03-2024.03.02
Contact person and Telephone number	Sun Zheng<br>18066061091
The signature of chairman	Zhu Zhen Fu
Ethics Committee	(Seal)
Approval Date	March 3rd ,2023
