# Supplementary material for: Moxibustion for declined cardiorespiratory fitness of apparently healthy older adults: A study protocol for a randomized controlled trial
Source: PLoS One. 2024 Apr 9;19(4):e0301673. doi: 10.1371/journal.pone.0301673 (PMC11003611; doi:10.1371/journal.pone.0301673)
Supplement: S1 Protocol — (PDF) [file pone.0301673.s003.pdf]

Clinical Research Implementation Plan

Project Number: ZT202208

Project Source: Jiangsu Provincial Administration of Traditional Chinese Medicine

Project Title: Clinical protocol quantification and optimization study of moxibustion to improve cardiorespiratory endurance in the elderly

Principal Investigator: Zhang Jianbin

Department Involved: Acupuncture and Moxibustion Department

Phone Number: 13851407748

Research Duration: January 2023 - December 2025

Version Date: February 16, 2023

Version Number: V1.0

Abstract

Title: "Clinical protocol quantification and optimization study of moxibustion to improve cardiorespiratory endurance in the elderly"

Objective: To observe the clinical effects of moxibustion in improving cardiorespiratory fitness in apparently healthy older adults; to apply and promote the clinical protocol of moxibustion for tonifying Qi and blood, improving cardiorespiratory fitness in older adults, effectively assisting older adults in maintaining health, improving quality of life, and contributing to elderly care.

Study Design: Randomized controlled trial.

Participants: Older adults from neighboring communities of the second affiliated hospital of Nanjing University of Chinese Medicine, Jianye District, Nanjing, China.

Sample Size: A total of 105 participants for the study on the clinical effects of moxibustion in improving cardiorespiratory fitness, with 35 participants in the moxibustion group, sham moxibustion group, and blank control group, respectively.

Treatment Protocol: (1) Moxibustion group: Moxibustion on bilateral Zusanli (ST36), Shenque (CV8), and Guanyuan (CV4) acupoints, 60 minutes per acupoint, once daily, five times a week (with weekends off), for a total of 12 weeks; (2) Sham moxibustion group: Utilization of specially modified moxibustion device to isolate the heat and radiation of moxa burning. Acupoint selection, moxibustion time, and frequency are the same as the moxibustion group; (3) Blank control group: No interventions given.

Inclusion Criteria: (1) Age between 60-80 years old. (2) Normal cognition and ability to complete all assessments and treatments. (3) CPET test indicating impaired cardiorespiratory fitness (VO_{2peak} < 20ml·kg-1·min-1 or 6MWD ≤ 450 meters).

Primary Outcome Measures: (1) Peak Oxygen Uptake (VO_{2peak}), (2) Anaerobic Threshold (AT), and (3) serum central carbon metabolites (CCB), including thirty-four CCB-related biomarkers such as adenosine triphosphate (ATP), nicotinamide adenine dinucleotide (NAD+), pyruvic acid, and acetyl-coenzyme A (Acetyl-CoA).

Secondary Outcome Measures: (1) 6-minute walk distance (6MWD), (2) the Short Form 36 Health Survey (SF-36), and the Qi and Blood Status Questionnaire (QBSQ).

Safety Measures: During CPET: (1) a drop in blood pressure exceeding the baseline static blood pressure by 20mmHg; (2) pathological Q waves or severe arrhythmias on electrocardiogram (ECG). (3) Severe hypertensive reaction (e.g., systolic blood pressure ≥220mmHg). During 6MWT: (1) a decrease in SpO_2 below 85% and sustained; (2) a decrease in systolic blood pressure of ≥20mmHg with an increased heart rate; (3) systolic blood pressure ≥180mmHg or diastolic blood pressure ≥100mmHg. Adverse events related to moxibustion treatment.

Statistical Analysis: The primary efficacy indicators will be analyzed using the full analysis set and data collected according to the protocol.

Clinical Research Project Implementation Plan for Moxibustion Improving Cardiorespiratory Fitness in Older Adults

1. Research Background and Basis

According to the requirements of the Jiangsu Provincial Administration of Traditional Chinese Medicine's scientific development project (special topic), this project aims to conduct clinical research on the quantification and optimization of the moxibustion protocol for improving cardiorespiratory fitness in elderly individuals.

(1) Domestic and International Research Status and Analysis

In May 2021, the National Bureau of Statistics released the results of the seventh national population census, revealing that the proportion of elderly people not only surpassed 10% for the first time (reaching 13.5%) but also increased by 4.63 percentage points, indicating an accelerated aging population. It is estimated that during the "14th Five-Year Plan" period, the total population of individuals aged 60 and above will exceed 300 million, accounting for over 20%, marking the stage of moderate aging. Around 2035, the population of individuals aged 60 and above will surpass 400 million, accounting for over 30% of the total population, entering a severe stage of aging. The aging population issue has once again attracted attention across various sectors in China. In the 2022 "Government Work Report," actively addressing population aging, optimizing elderly care services in urban and rural areas, and promoting the high-quality development of the aging industry were highlighted as national strategies. Improving the physiological condition of the elderly and maintaining their health not only enhances their quality of life but also reduces the socioeconomic burden on healthcare.

(2) Declining Cardiorespiratory Fitness in the Elderly Population

Cardiorespiratory fitness (CRF), also known as "overall fitness," is the body's capacity to intake, transport, and utilize oxygen during a specific workload and is regarded as the most crucial aspect among various indicators of physical health. It is considered the "fifth vital sign" in assessing an individual's overall health status[1]. Generally, higher levels of cardiorespiratory fitness correlate with better health conditions. However, survey data on populations aged 60 and above indicate varying degrees of decline in cardiorespiratory fitness[2,3].

From a functional perspective, cardiorespiratory fitness is primarily associated with the functions of the respiratory system, cardiovascular system, and the overall muscular system. The decline in cardiorespiratory fitness among the elderly is directly related to the decline in cardiorespiratory function. With advancing age, the myocardial cells of the elderly gradually atrophy, leading to decreased cardiac contractility and relaxation,

resulting in reduced cardiac function. Structural and functional changes in the respiratory system, such as thinning of alveolar walls, enlargement of alveoli, reduced number of pulmonary capillaries, decreased elasticity of lung tissues, and decreased respiratory muscle function, also contribute to the decline in cardiorespiratory fitness. Moreover, insufficient blood circulation and oxygen utilization in the body are related to factors such as systemic arterial atherosclerosis in the elderly.

The decline in cardiorespiratory fitness is closely linked to deficiency in Qi and Blood in the context of Traditional Chinese Medicine (TCM). Qi and Blood are fundamental substances in the human body's composition and the maintenance of vital life activities. Therefore, the decline in cardiorespiratory fitness is predominantly manifested as a state of deficiency of Qi and Blood and stasis of Qi and Blood.

(3) Declining Cardiorespiratory Fitness and its Relation to Various Chronic Diseases and Immune Aging

Apart from age-related decline, reduced cardiorespiratory fitness is associated with various chronic diseases, lifestyle habits, physical activity, etc. Studies[4,5] indicate that not only do patients with chronic obstructive pulmonary disease, coronary artery atherosclerotic heart disease, and hypertension experience severe declines in cardiorespiratory fitness, but individuals with metabolic disorders such as diabetes, hyperlipidemia, and osteoporosis, among others, also commonly exhibit decreased cardiorespiratory fitness. Therefore, the decline in cardiorespiratory fitness is both a cause and effect of chronic diseases, accelerating and aggravating their progression, which not only increases the socioeconomic burden but also severely impacts the quality of life of the elderly.

The innate and adaptive immune systems undergo age-related changes and gradually decline with aging, termed immunosenescence. For instance, natural killer (NK) cells in humans undergo significant differentiation with aging[6]. The decline in cardiorespiratory fitness in the elderly also implies an accelerated process of immune aging. The aging immune system reciprocally contributes to the overall aging of the body. Quantification of immunosenescence indicators, such as circulating leukocyte transcriptome analysis, assists in evaluating the body's immune function status, hence becoming a critical target for extending health span and delaying aging.

(4) Moxibustion, which tonifies Qi and nourishes blood, contributes to improving cardiorespiratory fitness

Currently, the primary strategy and method for enhancing and providing cardiorespiratory fitness primarily involve aerobic exercise. This approach is more suitable for athletes and younger individuals. However, for elderly individuals, there exists substantial individual variability in the intensity threshold for aerobic exercise. It becomes challenging to control exercise intensity and volume, thereby inevitably increasing the risk of exercise-related accidents. Hence, there is a necessity to explore

methods that are suitable for improving cardiorespiratory fitness in accordance with the physical characteristics of elderly individuals. Among these, the traditional Chinese medicine technique of moxibustion warrants deeper exploration.

Studies indicate that moxibustion, by warming and promoting circulation, not only facilitates the unblocking of meridians and regulates the circulation of Qi and blood but also warms and nourishes Qi and blood, meeting the needs of the body's organ tissues for Qi and blood[7]. Experimental research demonstrates[8]: moxibustion can prolong exhaustion time in normal rats during exercise; it can alleviate damage to tissues and cells in the liver, heart, skeletal muscles, kidneys, etc., caused by high-intensity exercise, thus alleviating fatigue. Additionally, moxibustion can elevate muscle glycogen and liver glycogen levels in rats during exercise training, ensuring the supply of glucose to tissues such as nerves and muscles during high-intensity exercise, thereby enhancing physical performance. Research on moxibustion's ability to enhance endurance and fatigue resistance demonstrates the clinical value and potential advantages of moxibustion techniques in improving and enhancing cardiorespiratory fitness.

(5) Enhancing cardiorespiratory fitness can improve immune function and enhance the health status of elderly individuals

Given the critical value of cardiorespiratory fitness in maintaining health, contemporary clinical practice is gradually focusing on the cardiorespiratory fitness of elderly individuals. Studies have shown a significant correlation between immune levels and cardiorespiratory function, with immune factor levels positively correlating with cardiorespiratory fitness[9]. Improving cardiorespiratory fitness not only aids in enhancing a patient's immune function but also benefits the recovery of cardiovascular and metabolic diseases, and osteoporosis, among others[10]. Therefore, it is essential to intensify attention and research on cardiorespiratory fitness in elderly individuals, particularly in identifying suitable techniques and methods to improve and enhance cardiorespiratory fitness, elucidating their role and value in anti-immune aging, enhancing immune function, and chronic disease rehabilitation.

(6) Moxibustion Enhancing Cardiorespiratory Fitness Needs Quantification and Optimization

The role of moxibustion in reducing fatigue and improving fitness has substantial evidence in normal individuals, athletes, and lower limb arterial disease patients. The preliminary confirmation of moxibustion improving cardiorespiratory fitness exists. However, there is inconsistency in clinical protocols for improving cardiorespiratory fitness through moxibustion, mainly in terms of acupoint selection and technical parameters for moxibustion. Currently, the commonly used acupoints for moxibustion to combat fatigue and increase fitness are primarily Shenque, Guanyuan, and Zusanli. As for moxibustion parameters, they vary widely, with moxibustion sessions ranging from 1 to 3 times per week and session durations ranging from 0.5 to 1 hour. Hence, in

the clinical protocol for moxibustion to improve cardiorespiratory fitness, it is necessary to study and define suitable technical parameters based on precise assessments to achieve quantification and optimization of the clinical protocol.

Given the progress of domestic and international research, it is believed that there is a widespread decline in cardiorespiratory fitness among the elderly, which is potentially associated with various chronic diseases and immune aging. Moxibustion, with its ability to warm meridians, and regulate Qi and Blood, may enhance cardiorespiratory fitness and immune function in elderly individuals. Conducting empirical research on moxibustion clinical protocols based on an objective analysis of cardiorespiratory fitness can further quantify and optimize these protocols. This endeavor aims to provide technical specifications for the promotion and application of moxibustion in tonifying Qi and Blood, improving cardiorespiratory fitness in the elderly, and enhancing immunity.

----

2. Research Objectives

The primary goal is to apply and promote the clinical protocol of moxibustion for tonifying Qi and Blood, thereby enhancing cardiorespiratory fitness in elderly individuals. The aim is to effectively assist the elderly in maintaining health, improving their quality of life, and contributing to elderly care initiatives. This study intends to observe the variations in primary outcome indicators (VO_{2peak}, AT value, serum central carbon metabolites, 6MWD, etc.) and adverse events occurring under the intervention of moxibustion. It aims to evaluate its effectiveness and safety.

----

3. Experimental Design Type, Principles, and Procedure

(1) Experimental Design Type: Randomized Controlled Trial

(2) Principles of Experimental Design:

Sample Size Calculation:

The sample size was determined based on our prior pilot investigation. A mean difference in VO2peak of 2.6 ml·kg-1·min-1, with a standard deviation (SD) of 3.0 was observed between the moxibustion and control groups. The estimation conducted via the website (https://sample-size.net/sample-size-means/). Approximately 29 participants each group are needed to detect a clinically significant difference in outcome measures at a 5% significance level and 90% statistical power. Considering an estimated dropout rate of 15%, the final sample size required will be 35 for each group, resulting in 105 participants. To recruit this number of participants, we anticipate a study period of 9 months based on our pilot study.

Randomization: Stratified Random Allocation

Control: The study includes apparently healthy elderly individuals, with no underlying diseases. Based on recognized principles of effectiveness, safety, and comparability,

sham moxibustion and no intervention are chosen as controls.

----

4. Participant Selection

This study will recruit participants from the neighboring community of the Second Affiliated Hospital of Nanjing University of Traditional Chinese Medicine through brochures, posters, and community outreach programs.

(1) Inclusion Criteria:

- Men or women aged between 60 and 80;
- No cognitive impairment;
- Decreased CRF ($VO_{2peak} < 20$ml·kg-1·min-1 or 6MWD ≤ 450 meters).

(2) Exclusion Criteria:

- Acute stage of serious diseases, such as acute myocardial infarction, acute myocarditis, unstable angina;
- With any other absolute contraindications to CPET or the Six-minute walk test (6MWT);
- With any underlying diseases, such as hypertension, diabetes, coronary heart disease;
- Currently using other Chinese and Western medicines that affect the results of this study;
- Other physical injuries that affect the evaluation of observation indicators, such as lower extremity pain, nerve and muscle diseases;
- Respiratory symptoms within one month;
- Other conditions that may reduce the possibility of enrollment or complicate enrollment, such as frequent changes in the living environment, are likely to cause a loss of follow-up.

(3) Dropouts:

- Participants experiencing severe adverse events, as judged by the physician, necessitating termination of their clinical trial participation.
- Occurrence of significant deviations during the implementation of the clinical trial protocol, such as poor compliance, difficulties in evaluating moxibustion efficacy, etc.
- Significant lifestyle changes including but not limited to alterations in diet, and exercise habits.
- Weight fluctuation of more than 5kg.
- Participants express unwillingness to continue the clinical trial during the trial process and request to withdraw from the study under the overseeing physician.
- Unforeseen circumstances hindering the participant's involvement, such as severe accidents, sudden serious illnesses, or death.

(4) Handling of Dropouts:

Dropout Criteria: Participants who, after providing informed consent and being screened as eligible for randomization, fail to complete the specified treatment or observation period according to this protocol will be considered dropout cases.

Handling of Dropouts:

- Researchers should attempt to contact participants who drop out through home visits, scheduled follow-ups, phone calls, letters, etc., to inquire about the reasons for withdrawal, record the last intervention time, and complete as many assessment items as possible.
- In case of dropout due to allergies, adverse reactions, or ineffective treatment, appropriate treatment measures should be taken based on the participant's actual condition.
- All information related to dropout cases should be properly preserved for archival purposes and for statistical analysis in the comprehensive dataset. No additional recruitment of dropout patients is necessary.

(5) Exclusion of Cases:

- Cases that do not meet the inclusion criteria but fulfill exclusion criteria.
- Cases that have not used the experimental intervention.
- Cases without any data after randomization.
- Before statistical data analysis, the decision to exclude cases will be made after discussion among statisticians and principal investigators.

----

5. Treatment Plan

(1) Equipment Used for the Trial

Type and Specifications: YKY-B.

Moxa sticks: Specifications: 100mm×270mm, provided by Shanxi Yingkaiyuan Biotechnology Co., Ltd.

Moxibustion equipment: Moxa tubes, moxibustion base, fixing adhesive tape, provided by Shanxi Yingkaiyuan Biotechnology Co., Ltd.

(2) Intervention Methods

Moxibustion Group:

Selection of Acupoints: Bilateral Zusanli, Shenque, Guanyuan points.

Acupoint Location: Following the 2006 National Standard of the People's Republic of China "Names and Locations of Acupoints" (GB/T12346-2006): the ST36 is located at 3 cun (about 10 cm) below the patella, outside of the anterior crest of the tibia. CV8 is located at the navel, and CV4 is 3 cun directly below the CV8.

Moxibustion Operation:

- Patient lies supine, exposing the skin at the selected acupoints, hands placed on either side of the body.

- The practitioner affixes the moxibustion device base using adhesive tape on the selected acupoints.
- Ignite the moxa stick on the moxibustion cylinder.
- Position the moxibustion device cylinder so that its top is level with the bottom of the device base.
- Rotate the moxibustion cylinder to adjust the air outlet size, maintaining a distance of approximately 3cm from the skin; make participants feel comfortably warm without burning pain.
- When the warm feeling diminishes, indicating the moxa stick is burnt out, it can be replaced.
- After 60 minutes of moxibustion, close the air outlet, remove the moxibustion device, and place the extinguished moxa stick in a container with water.
- Check the moxibustion device for damage and store it back in the original packaging for future use.
- Each participant undergoes moxibustion once a day (8:00-12:00 AM or 2:00-5:00 PM), 60 minutes per acupoint, five times a week (with weekends off), 12 weeks (60 sessions) in total.

Sham Moxibustion Group:

Selection of Acupoints: Same as the Moxibustion Group.

Acupoint Location: Same as the Moxibustion Group.

Shame Moxibustion Operation:

- Patient lies supine, exposing the skin at the selected acupoints, hands placed on either side of the body.
- The practitioner affixes the modified moxibustion device base (equipped with a thermal insulating metal membrane) using adhesive tape on the selected acupoints.
- Ignites the moxa stick on the moxibustion cylinder.
- Positions the moxibustion device cylinder so that its top is level with the bottom of the device base.
- Rotates the moxibustion cylinder to adjust the air outlet size, maintaining a distance of approximately 5cm from the skin.
- Replace the moxa stick after 30 minutes.
- After 60 minutes of moxibustion, close the air outlet, remove the moxibustion device, and place the extinguished moxa stick in a container with water.
- Check the moxibustion device for damage and store it back in the original packaging for future use.
- Each participant undergoes moxibustion once a day (8:00-12:00 AM or 2:00-5:00 PM), 60 minutes per acupoint, five times a week (with weekends off), 12 weeks (60 sessions) in total.

Blank Control Group:

The blank control group will receive no treatment and will be instructed not to undergo any interventions that might affect cardiorespiratory fitness until the trial is completed. Measurements for the blank control group will follow the designated time points as the Moxibustion and Sham Moxibustion Groups.

----

6. Assessments

(1) General Recording Items

Participant code, initials of the participant's name in Pinyin, and trial start date.

(2) Observation Indicators

- Biological Indicators: Demographic Characteristics: Gender, age, height, weight.
- Vital Signs: Body temperature, resting heart rate, respiration, blood pressure (systolic and diastolic) after 10 minutes of rest.

(3) Diagnostic Indicators

- CPET Results: Peak Oxygen uptake (VO_{2peak}).
- Six-Minute Walk Test Results: Distance covered in six minutes (6MWD).

(4) Outcome measures

- **Primary Outcome Measures: Peak Oxygen Uptake (VO_{2peak}) and Anaerobic Threshold (AT)**

The assessment process is as follows:

The CPET procedure involves the following steps: ① Participant completes a health screening questionnaire; ② Participant wears loose, comfortable exercise attire, avoids heavy meals or prolonged fasting before testing, refrains from smoking or consuming strong coffee, and prepares a cup of water; ③ Guidelines and precautions are provided for the evaluation; ④ Participant familiarizes themselves with the exercise protocol, equipment, and pedal speed (50-60r/min), wears a mouthpiece and nose clip to prevent air leakage, adjusts the exercise bike's seat and handlebar height to the most suitable position, becomes acquainted with the Borg Rating of Perceived Exertion scale, common reasons for stopping exercise, and non-verbal communication methods when feeling significantly uncomfortable during exercise; ⑤ The test physician assesses the participant, selects an appropriate exercise power scheme; ⑥ Execution of the scheme under the guidance and supervision of the test physician; ⑦ After the test, the assessing physician extracts and records the participant's VO2peak and AT value for subsequent data analysis.

Considering the physiological characteristics of older adults and to ensure the completeness and smoothness of the experiment, the trial plans to use the Ramp scheme for the power bike to increment the load. The specific method is as follows: Calculate according to the formula: Predicted unloaded VO_2 value (predicted unloaded $VO_2 = 150 + [6 \times \text{body weight (kg)}]$) and predicted VO_{2peak} ([height (cm) - age] × 20 (males) or × 14 (females)). It is generally recommended to complete the incremental exercise test

within 6-10 minutes. If completed in 10 minutes, the increase in power (W) per minute = [predicted VO_{2peak} - predicted unloaded VO_2]/100. However, the power increment speed is not fixed. For older adults with low daily activity levels or underlying cardiovascular or respiratory system diseases, consider reducing the power increment range.

The specific testing procedure includes: ① Resting phase (3 minutes): Obtain resting heart rate, blood pressure, electrocardiogram, static gas metabolism, and other indicators; ② Unloaded warm-up exercise phase (3 minutes): Maintain a cycling speed of 55-65 r/min, record heart rate, blood pressure, electrocardiogram, blood oxygen saturation, etc., close to the end; ③ Power load phase (6-10 minutes): Maintain cycling speed at 55-65 r/min, closely observe participant's symptoms, heart rate, blood pressure, gas metabolism indicators, blood oxygen saturation, and changes in the electrocardiogram. Note potential adverse reactions during the test, such as angina, breathing difficulties, dizziness, paleness, sweating, significant rapid or slow arrhythmias, ischemic changes in the electrocardiogram, blood pressure drop, shock, etc. If any of these symptoms or signs appear during exercise, the test should be immediately terminated, and appropriate observation and treatment should be given; ④ Recovery phase (6-8 minutes): Maintain slow cycling without load for 2-3 minutes, cycling speed can be maintained at 30-40 r/min, observe the participant's heart rate, blood pressure, electrocardiogram, and symptoms. Stop cycling without load, and continue observing whether the participant's vital signs such as heart rate and blood pressure return to pre-test levels. If symptoms and/or abnormal signs persist for more than 15 minutes after terminating exercise, further observation or treatment is required.

- **Primary Outcome Measures: Serum Central Carbon Metabolites (CCB)**

The main steps of the detection process include sample collection, target metabolite extraction, standard curve establishment, LC-MS/MS detection, and data analysis.

① Instrumentation used: AB Sciex QTRAP® 6500+ Mass Spectrometer, AB Sciex Exion LC™ Liquid Chromatography. ② Reagents: 34 central carbon-related substance standards and 5 stable isotope-labeled standards purchased from Shanghai Zhenzhun Biotechnology Co., Ltd.; Methanol, Acetonitrile, Formic acid, Ammonium acetate (LC-MS, Thermo-Fisher, USA), Aminomethanediphosphonic acid (Sigma-Aldrich, USA), Milli-Q ultrapure water (Millipore, USA). ③ Sample collection: Participants fasted and venous blood was collected in the hospital laboratory. Blood was allowed to clot at room temperature in a centrifuge tube or vacuum blood collection tube for 1 hour. Then centrifuged at 3000rpm for 10 minutes, the supernatant was transferred into 1.5mL centrifuge tubes (0.2mL per tube). After labeling, samples were rapidly frozen in liquid nitrogen for 15 minutes and stored at -80°C. ④ Preparation of standard solutions: Accurately weighed 34 central carbon-related substance standards to prepare a mixed standard solution with a concentration of 100μg/mL. Methanol was used to dilute the

linear mother liquor to obtain working solutions of various concentrations. Solutions of D-Glucose-13C6, (s)-Malicacid-D3, Succinic acid-D4, dAMP lithium salt-15N5, and D-Glucose-6-phosphate disodium salt-13C6 were prepared at certain concentrations and mixed to obtain an internal standard solution (IS). Mother liquor and working solutions for linearity, internal standards, and quality control were stored at -20°C. Metabolite extraction: Take 100mg of sample and add 500μL of mixed internal standard solution (methanol: water = 4:1). Mix well, stand for 5 minutes, centrifuge at 12000rpm for 10 minutes, and take the supernatant for LC-MS analysis. ⑤ Chromatographic and mass spectrometric methods: Chromatographic column: Waters Atlantis Premier BEH Z-HILIC (2.1×100mm, 1.7μm); Mobile phase: Phase A: 15mM Ammonium acetate + 10μm Aminomethanediphosphonic acid aqueous solution; Phase B: 15mM Ammonium acetate/Acetonitrile; Column temperature: 50°C; Injection volume: 2μL; Flow rate: 0.4 mL/min. Mass spectrometry conditions: Electrospray ionization (ESI), negative ionization mode. Ion source temperature at 550°C, ion source voltage at -4500V, curtain gas at 35psi, and both nebulizer gas and auxiliary gas at 60psi. Multiple reaction monitoring (MRM) was used for scanning.

- **Secondary Outcome Measures: Six-Minute Walk Test (6MWT)**

The test site and standardized execution criteria are as follows: ① Test Site Setup: Select a long, straight, flat, hard-surfaced corridor, 2-3 meters wide, and 30 meters long, with markings every 3 meters. A brightly colored strip is placed on the ground as the start and finish line, with a prominent traffic cone at the turning point. ② Test Physician Preparation: Preparation includes a timer, lap counter, pulse oximeter, blood pressure cuff, test record sheet, portable chair, etc. ③ Participant Preparation: Participants wear loose, comfortable athletic shoes, socks, and clothing. They should avoid heavy meals or prolonged fasting before the test. During the test, participants can use their daily walking aids, such as canes or walkers. ④ Explanation to Participants: The test physician explains the purpose, principles, and procedures of the 6MWT to the participants, including information about taking breaks midway and resuming the test. ⑤ Participant Readiness: Before the test, participants rest on a chair near the starting point for at least 15 minutes. The physician checks for potential contraindications, collects basic information on the test form, measures arterial blood pressure, heart rate, and oxygen saturation, and evaluates the patient's baseline dyspnea and fatigue using the Borg scale. ⑥ Test Initiation: Participants begin the test, and the physician guides, encourages, reminds, and monitors participants at fixed time intervals using standardized verbal cues. ⑦ Complete record of test data, including participant information, actual distance covered in the 6MWT (in meters), percentage of the actual distance to the predicted value, heart rate, blood pressure, oxygen saturation, and any stops or rest taken during the test.

- **Secondary Outcome Measures: the Short Form 36 Health Survey (SF-36)**

The Short Form 36 Questionnaire (SF-36) is used to assess the quality of life in elderly individuals. The SF-36 is a concise health survey that comprehensively covers eight aspects of participants' quality of life, including physical functioning, physiological role limitations, bodily pain, general health perceptions, vitality, social functioning, emotional role limitations, and mental health. Portions related to physical functioning, physiological role limitations, vitality, etc., directly reflect the impact of changes in cardiorespiratory fitness on the quality of life in elderly individuals.

Scoring Method for SF-36:

Converted Score = (Actual Score - Lowest Possible Score for the Aspect) / (Difference between the Highest and Lowest Possible Scores for the Aspect) X 100

Unanswered items are considered missing. The score for missing items is replaced with the average score for the relevant aspect.

- **Secondary Outcome Measures: the Qi and Blood State Questionnaire (QBSQ)**

The Traditional Chinese Medicine Constitution Scale is a standardized questionnaire used to identify traditional Chinese constitutions. Developed collaboratively by the China Academy of Chinese Medical Sciences and Beijing University of Chinese Medicine, this questionnaire evaluates nine types of constitutions, including Qi Deficiency, Blood Stasis, and Qi Stagnation. This study uses Qi Deficiency, Blood Stasis, and Qi Stagnation scales to determine participants' Traditional Chinese Medicine Qi and Blood states.

Specific evaluation process: ① Selection of Assessors: Clinical experienced TCM practitioners with a foundation in TCM theories are chosen as assessors. ② Questionnaire Completion: Assessors question participants based on items in various scales, and participants choose the most suitable answers based on personal experiences and feelings. ③ Score Calculation: Based on the completed questionnaire, assessors calculate the direct and converted scores for participants in different constitutional types. They then determine whether the participant's constitution is "Yes," "Tendency Yes," or "No" based on the scores.

Scoring Method for Traditional Chinese Medicine Constitution Scale:

Raw Score = Sum of Scores for Each Item

Converted Score = ((Raw Score - Number of Items) / (Number of Items × 4)) × 100%

Constitutional judgment based on the converted score: "No" for scores <30, "Tendency Yes" for scores between 30-39, and "Yes" for scores ≥40.

(4) Safety Observation

- During cardiorespiratory exercise tests, if any of the following danger signs appear before the patient reaches symptomatic limits, consider terminating exercise: central nervous system symptoms like dizziness, vertigo, etc. If blood pressure decreases instead of increasing during exercise, dropping more than 20mmHg from

baseline systolic blood pressure. Pathological Q waves or severe arrhythmias on electrocardiogram, such as multiple frequent ventricular arrhythmias. Severe hypertensive response (e.g., systolic blood pressure ≥220mmHg).

- During the 6MWT, consider stopping the test if any of the following danger signs appear: suspected angina with chest pain; intolerable shortness of breath; leg spasms or extreme leg muscle fatigue; gait imbalance; pallor, sweating; dizziness or fainting; SpO2 consistently below 85%; systolic blood pressure drops ≥20mmHg with an increased heart rate; systolic blood pressure ≥180mmHg or diastolic blood pressure ≥100mmHg; inability of the participant to continue the test.
- During moxibustion treatment, observe and record any allergic reactions or burn injuries that occur during or after treatment.

(5) Trial Evaluation Indicators

- Concomitant Medications: Record the medications taken by participants during the trial, especially those that may influence the study results, such as beta-blockers.
- Dropout and Exclusion Rate Assessment: Detailed statistics on the number, time, assessment status, and reasons for dropout or exclusion during the trial, along with calculation of dropout and exclusion rates.
- Compliance Evaluation: Record the actual number of times participants received moxibustion treatment during the trial. Calculate the ratio of actual treatment received to planned treatment, for compliance assessment.

(6) Observational Time Points

- After participants are enrolled, collect basic information (general information, dietary habits, exercise habits, etc.) and baseline measures, including VO_{2peak}, AT, serum CCB, 6MWD, SF-36, and QBSQ;
- After the 4th and 8th weeks of treatment, conduct and record twice interim assessments, including VO_{2peak}, AT, serum CCB, 6MWD, SF-36, and QBSQ;
- At the end of the treatment (12 weeks), collect basic information again (general information, dietary habits, exercise levels, etc.) and outcome indicators, including VO_{2peak}, AT, serum CCB, 6MWD, SF-36, and QBSQ;
- Follow-up until the 24th week, evaluating indicators including VO_{2peak}, AT, 6MWD, SF-36, and QBSQ.

----

7. Observation of Adverse Events

(1) Definition

The term "adverse event" encompasses any symptom, syndrome, or disease occurring in patients during clinical research that affects patient health. This also includes clinically relevant situations identified in the laboratory or other diagnostic procedures, requiring unplanned diagnostic or therapeutic measures, resulting in withdrawal from

the study, or showing clinically significant abnormal laboratory test results.

Adverse events may be new diseases; worsening of symptoms or signs related to the treatment status or the disease itself; unrelated to participation in the trial; or a combination of one or more factors. Therefore, the term "adverse event" does not necessarily imply a causal relationship with the trial therapy.

A serious adverse event refers to adverse events occurring at any dose of the study treatment regimen or at any time during the observation period, including those resulting in death; immediate life-threatening situations; hospitalization or prolonged hospital stay; permanent or severe disabilities; overdose-related events; causing cancer; congenital abnormalities; of significant medical importance (referring to events that do not immediately endanger life or cause death or require hospitalization but may harm patients or require measures to prevent one of the defined consequences); requiring medical intervention to prevent permanent damage or harm.

(2) Expected Adverse Reactions in the Trial

Although preclinical data has not shown adverse reactions, attention should still be paid to potential local blisters, itching, and rash reactions.

(3) Causality Assessment of Adverse Events with Drugs

According to the standards set by the National Center for Adverse Drug Reaction Monitoring of the Ministry of Health (see "Guidelines for Clinical Research of New Traditional Chinese Medicine (Trial)"), an assessment of the possible relationship between adverse events and investigational drugs is made using a five-level classification system: "certain, probable, possible, suspected, unlikely." Assessment criteria can be found in the "Adverse Event Form" in the study medical records.

(4) Management of Adverse Events

- Observation and Recording: Investigators should request patients to truthfully report changes in their condition after drug administration and avoid leading questions. Any adverse reactions occurring during the trial should be recorded on the "Adverse Event Form," and investigations should be conducted, detailing the process and results of handling until laboratory tests return to normal and symptoms disappear. The follow-up method can vary from hospitalization, outpatient visits, home visits, phone calls, correspondence, etc.
- Medical Care: When adverse reactions are discovered, investigators decide on diagnosis and treatment measures based on the condition, and determine whether to discontinue observation. In the case of serious adverse events, the unit conducting the clinical research must take necessary measures immediately to ensure the safety of participants.
- Reporting: Investigators complete the "Serious Adverse Event Report Form" and report to the department, medical office, and ethics committee within 24 hours, signing and indicating the date on the report.

----

8. Assessment Criteria for Efficacy and Safety

(1) Comprehensive Efficacy Assessment Criteria: Statistical differences in the main outcome indicators are used to judge efficacy.

(2) Criteria for Severity of Adverse Events:

- Mild: Tolerable for participants, does not affect treatment, requires no special treatment, and has no impact on participants' recovery.
- Moderate: Difficult for participants to tolerate, requires withdrawal of drugs to stop the trial or special treatment, and has a direct impact on the participants' recovery.
- Severe: Endangers the participants' lives, causes death or disability, requires immediate drug withdrawal, or urgent treatment.

----

9. Quality Control and Assurance of the Trial

(1) Laboratory Quality Control Measures

- Establish standard operating procedures and quality control procedures for experimental observation indicators.
- Laboratory test results must be printed by a computer and the data should be traceable.

(2) Standard Operating Procedures for Main Observation Indicators

Strictly adhere to relevant guidelines, consensus, and standards for each evaluation item, such as the CPET examination mainly refers to the "Adult Cardiopulmonary Exercise Testing Guideline"[11]; Implementation of the Six-Minute Walk Test refers to the AST2002 version guideline[12].

(3) Pre-trial Clinical Training

Training researchers on the trial protocol before the project starts and ensuring consistency in quantifying symptoms and signs. Researchers must sign a declaration.

(4) Measures to Improve Participant Compliance

- Timely reminders for follow-up visits.
- Waiver of all treatment and assessment costs.
- Providing financial compensation to participants who complete all planned treatments and assessments.

(5) Quality Control and Quality Assurance System

Researchers must fulfill their respective responsibilities and strictly adhere to the clinical trial protocol using standard operating procedures to ensure the implementation of quality control and quality assurance systems in clinical trials.

----

10. Data Management

(1) Data Recording

- Research records are the source documents of clinical trial participants and are kept

in the office of the director of the Acupuncture and Moxibustion Department at the hospital.

- Requirements for research record keeping: ① Researchers must write research records concurrently while treating participants to ensure timely, complete, accurate, and truthful data recording. ② Any evidence-based corrections in the research records can only be crossed out, and changes noted by the researcher's signature and date, without erasing or covering the original records. ③ Participants' original test reports are pasted into the research records.

(2) Data Reporting

- Research records.

(3) Data Monitoring

The Ethics Review Committee of the Second Affiliated Hospital of Nanjing University of Chinese Medicine reviews each research record.

(4) Data Verification

- Establish an electronic database using Microsoft Excel software.
- Conduct numerical range and logic checks. In case of any doubts, fill out a query list. Researchers will address the issues listed in the query list. After completing the data verification report, lock the database.
- Relevant files about quality control should be saved, such as original records of data consistency checks, numerical range and logic checks, original records during blind review, and records of queries exchanged among researchers.

----

11. Statistical Analysis

(1) Statistical Analysis Plan and Statistical Software

- Full Analysis Set: Refers to the ideal set of participants as close as possible to the intention-to-treat principle (mainly analyzing all randomized participants). This dataset is derived by excluding participants from all randomized participants in the least and reasonable manner. For missing values of the primary variables, carry-forward estimation will be used, bringing forward the results closest to a single observation of the missing data in the trial, and ensuring consistency between the number of participants evaluated for efficacy at the endpoint and those at the start of the trial.
- Statistical Analysis Contents: Actual number of enrolled participants in three groups, dropout and exclusion cases, demographic and other baseline characteristics, compliance, efficacy analysis, and safety analysis.

(2) Statistical Analysis Methods

Summarize the demographic characteristics of each group and baseline variable measurements. The Kolmogorov-Smirnov test will be used for assessing data normality. Continuous data will be represented as the mean ± standard deviation for normally

distributed data and median (range) for non-normally distributed data. Paired t-tests or analysis of variance (ANOVA) will be used to assess changes in primary and secondary outcomes before and after intervention. Analysis of variance or Kruskal-Wallis test will be used for pairwise comparison of primary and secondary outcomes among groups. Chi-square or Fisher's exact test will be used for analyzing categorical data. Pearson or Spearman correlation analysis will be used to assess the correlation between variables. Statistical analysis will be conducted using the Windows version of the Social Sciences Statistics Software Package (SPSS) 27.0, with significance set at a two-tailed P-value of <0.05. Graphical analysis will be performed using GraphPad Prism software (version 9.4.0).

----

12. Ethical Principles

(1) Ethical Review: The clinical trial protocol is implemented after approval by the ethics committee. If revisions are made during the trial, it must be re-submitted for approval by the ethics committee before implementation. If significant new information regarding the trial medication is discovered, the informed consent form must be modified in writing, submitted for approval by the ethics committee, and re-obtained from the participants.

This research project undergoes ethical review by the responsible unit's ethics committee before the trial commences. Each participating unit's ethics committee will be informed, and if necessary (such as the occurrence of serious adverse events), meetings should be promptly convened for review, and conclusions communicated to other central ethics committees.

(2) Benefits and Risks: Participants may benefit from this study. These benefits include potential improvements in participants' conditions and the potential development of a new treatment method for other patients with similar conditions. Participants will receive free medical examinations related to the study and registration fees during follow-ups. Participants will receive good medical care during the study.

(3) Recruitment of Participants: Recruitment of participants will be through internal notices posted, interested individuals applying, reading the research synopsis, signing the informed consent form, volunteer physical examinations, screening, and selection of eligible participants through random grouping. Recruitment notices and research synopses are included in the annex and submitted for ethical committee review.

(4) Medical Care and Protection of Participants: Researchers from each participating unit are responsible for the medical care of participants, making relevant medical decisions, and ensuring appropriate treatment if adverse events occur during the trial.

(5) Protection of Participant Privacy: Only research personnel involved in the clinical trial may access the participants' medical records. Data processing will be anonymized, omitting information that could identify individual participants.

(6) Informed Consent Process: Details about the clinical trial, including its purpose, procedures, potential benefits and risks, participants' rights, and obligations, will be explained. Participants will have ample time to consider and have satisfactory answers to their questions before consenting and signing the "Informed Consent Form." Each patient signing the informed consent form should be provided with the doctor's contact number to reach out in case of changes in their condition.

----

13. Summary and Data Preservation

(1) Summary and Conclusion

The responsible unit will complete the statistical work of the clinical trial data.

(2) Data Preservation

Research records (Case Report Forms), informed consent forms, records of moxibustion treatment, etc., will be archived and preserved.

----

14. Task Allocation, and Expected Progress

(1) Task Allocation

Research Design: Jianbin Zhang, Zheng Sun;

Research Investigation: Zheng Sun, Zhihong Xu, Kuang Yu, Haitian Sun;

Methodology: Zhihong Xu, Zheng Sun, Yiren Lin, Yimin Zhu;

Project Management: Zheng Sun, Jianbin Zhang;

Project Supervision: Jianbin Zhang;

Drafting of Research Proposal: Zheng Sun, Zhihong Xu, Zilong Zhu, Yiren Lin, Yimin Zhu, Jianbin Zhang.

(2) Expected Progress

Q1 2023: Development of research manuals, study plans, informed consent forms, and related materials.

Q2 2023: Ethical review, recruitment promotion, clinical trial registration.

Q3 2023: Initiate recruitment; draft and publish research proposals.

Q4 2023: Recruitment of participants, completion of preliminary screenings.

Q1 2024: Recruitment of participants, completion of preliminary screenings.

Q2 2024: End recruitment; inclusion and exclusion of participants.

Q3 2024: Stratified random grouping, completion of baseline data collection and recording.

Q4 2024: Commence acupuncture intervention and regular follow-ups.

Q1 2025: End intervention, follow-up on outcome indicators.

Q2 2025: Follow-up phase.

Q3-4 2025: Data analysis, and draft research report.

----

15. References

[1] Ross R, Blair SN, Arenar, et al. Importance of assessing cardiorespiratory fitness in clinical practice: a case for fitness as a clinical vital sign: a scientific statement from the American Heart Association [J]. Circulation, 2016, 134(24):e653-e699.
[2] Lavie CJ, Milani RV. Disparate effects of improving aerobic exercise capacity and quality of life after cardiac rehabilitation in young and elderly coronary patients[J]. J Cardiopulm Rehabil, 2000,20:235-240.
[3] Tang J, Li M H. Detection and application of physiological load of exercise intensity[J]. Chinese Journal of Tissue Engineering Research, 2012,16(20):3784-3788.
[4] Zhou L, Zhang F C, Chu X M, et al. Influence of cardiac rehabilitation on improvement rate of cardiorespiratory endurance in patients with coronary heart disease[J]. Journal of Clinical Electrocardiology, 2022,31(02):131-135.
[5] Zheng Y, Zou C Y, Wu L J, et al. Study on the correlation between cardiorespiratory endurance and body composition, metabolic indicators in middle-aged men with impaired fasting glucose[J]. Chinese and Foreign Medical Research, 2022,20(14):158-162.
[6] Matthew J, Yousefzadeh, Rafael R, et al. An aged immune system drives senescence and ageing of solid organs. Nature,2021,594(7861): 100–105.
[7] Zhang J B, Wang L L, Hu L, et al. Theoretical discussion on the warming and dredging effects of moxibustion[J]. Chinese Acupuncture, 2011,31(01):51-54.
[8] Li C R, Sun Z R, Wang Y L, et al. Clinical application and mechanism discussion of moxibustion in the treatment of chronic fatigue syndrome[J]. International Journal of Traditional Chinese Medicine, 2022,44(08):954-958.
[9] Xu S, Li S C. Study on the correlation between serum testosterone level, immune factors, and cardiorespiratory endurance in adolescent swimmers[C]//. The 12th National Sports Science Conference Paper Abstract Compilation——Poster Exchange (School Sports Sub-Committee).[Publisher unknown],2022:1230-1232.
[10] Zhang H P, Yi R, Gao Y. Effects of Tai Chi practice on cardiorespiratory endurance, blood glucose, and blood lipids in elderly male diabetic patients[C]//. The 11th National Sports Science Conference Paper Abstract Compilation, 2019:5476-5477.
[11] De Boer E, Petrache I, Mohning M P. Cardiopulmonary Exercise Testing [J]. Jama, 2022, 327(13): 1284-5.
[12] ATS Committee on Proficiency Standards for Clinical Pulmonary Function Laboratories. ATS statement: guidelines for the six-minute walk test. Am J Respir Crit Care Med,2002,166:111-117.

----

16. Abbreviations

CRF: Cardiorespiratory Fitness

CPET: Cardiopulmonary Exercise Testing

6MWT: 6-Minute Walk Test

VO_{2peak}: Peak Oxygen Uptake
AT: Anaerobic Threshold
SF-36: Short Form-36 Health Survey
QBSQ: the Qi and Blood State Questionnaire
