# Supplementary material for: Moxibustion for declined cardiorespiratory fitness of apparently healthy older adults: A study protocol for a randomized controlled trial
Source: PLoS One. 2024 Apr 9;19(4):e0301673. doi: 10.1371/journal.pone.0301673 (PMC11003611; doi:10.1371/journal.pone.0301673)
Supplement: Attachment — Submitted filename: Response to reviewers.docx [file pone.0301673.s004.docx]

**Responses to the editor**

**1. Please ensure that your manuscript meets PLOS ONE's style requirements, including those for file naming.**

**Our response:** Thank you for your kind reminder. We have carefully modified the formatting of our manuscript based on the PLOS ONE’s style requirements provided. The main adjustments include: **1)** adjusting the font size of the title and headings; **2）**using required symbols in the author and affiliation sections; **3)** relocating figure captions to follow the paragraphs where they are first mentioned; **4)** appending supporting information after the references; **5)** introducing line numbers, etc. We trust that these adjustments align with the journal’s specifications.

Besides, all figures have been uploaded to the Preflight Analysis and Conversion Engine (PACE) digital diagnostic tool, and we believe that all figures meet PLOS requirements.

**2. We note that the grant information you provided in the ‘Funding Information’ and ‘Financial Disclosure’ sections do not match.**

**When you resubmit, please ensure that you provide the correct grant numbers for the awards you received for your study in the ‘Funding Information’ section.**

**Our response:** We are very sorry for making such a mistake. We have reviewed and rectified the funding grant information. We uploaded a new “Funding statement” file to ensure it is consistent with the information in the “Financial Disclosure”.

**3.** **Your ethics statement should only appear in the Methods section of your manuscript. If your ethics statement is written in any section besides the Methods, please delete it from any other section.**

**Our response:** Thank you for this kind reminder. All ethical statement has been deleted from the manuscript **except for** the “Ethics statement and registry” under “Methods/design” section (**Line 96-101, Page 5**).

**4.** **We note that Figures 4 and 5 in your submission contain copyrighted images. All PLOS content is published under the Creative Commons Attribution License (CC BY 4.0), which means that the manuscript, images, and Supporting Information files will be freely available online, and any third party is permitted to access, download, copy, distribute, and use these materials in any way, even commercially, with proper attribution. For more information, see our copyright guidelines: http://journals.plos.org/plosone/s/licenses-and-copyright.**

**We require you to either (1) present written permission from the copyright holder to publish these figures specifically under the CC BY 4.0 license, or (2) remove the figures from your submission:**

**1. You may seek permission from the original copyright holder of Figures 4 and 5 to publish the content specifically under the CC BY 4.0 license.**

**We recommend that you contact the original copyright holder with the Content Permission Form (**[**http://journals.plos.org/plosone/s/file?id=7c09/content-permission-form.pdf**](http://journals.plos.org/plosone/s/file?id=7c09/content-permission-form.pdf)**) and the following text:**

**“I request permission for the open-access journal PLOS ONE to publish XXX under the Creative Commons Attribution License (CCAL) CC BY 4.0 (**[**http://creativecommons.org/licenses/by/4.0/**](http://creativecommons.org/licenses/by/4.0/)**). Please be aware that this license allows unrestricted use and distribution, even commercially, by third parties. Please reply and provide explicit written permission to publish XXX under a CC BY license and complete the attached form.”**

**Please upload the completed Content Permission Form or other proof of granted permissions as an "Other" file with your submission.**

**In the figure caption of the copyrighted figure, please include the following text: “Reprinted from [ref] under a CC BY license, with permission from [name of publisher], original copyright [original copyright year].”**

**2. If you are unable to obtain permission from the original copyright holder to publish these figures under the CC BY 4.0 license or if the copyright holder’s requirements are incompatible with the CC BY 4.0 license, please either i) remove the figure or ii) supply a replacement figure that complies with the CC BY 4.0 license. Please check copyright information on all replacement figures and update the figure caption with source information. If applicable, please specify in the figure caption text when a figure is similar but not identical to the original image and is therefore for illustrative purposes only.**

**Our response:** We really appreciate it and thank your reminder regarding this noteworthy issue. We proceed to elucidate and address the copyright concerns associated with Figures 4 and 5, respectively.

**Figure 4** (Title: Acupoints applied in the trial). We have replaced the original image with a human body picture captured by us. The individual in the replaced figure has provided consent and authorization for us to use the image in paper publication, allowing subsequent free downloading, copying, and distribution in any manner, including commercial purposes.

**Figure 5** (Title: Ignition-type moxibustion device). Based on photos of the moxibustion device to be used, we re-drew the line draft using software Adobe illustrator 2023 and replaced the original Figure 5 with the revised version.

We've updated the figure caption **(Line 170-172, Page 8 for Fig 4; Line 200-204, Page 10 for Fig 5)** for the alternate figures to specify its source to avoid any copyright issues.

**5.** **Please include captions for your Supporting Information files at the end of your manuscript, and update any in-text citations to match accordingly. Please see our Supporting Information guidelines for more information: http://journals.plos.org/plosone/s/supporting-information.**

**Our response:** Thank you for this friendly reminder. Supporting information files have been appended after the References section (**Line 548-554, Page 27**). Notably, these supplementary documents are not cited in the main text, so there are no updates to in-text citations.

**Responses to the reviewers**

**Reviewer #1:**

**This is a study protocol to evaluate the efficacy of Moxibustion in elderly. I have a few comments to the authors.**

**Our response:** We sincerely appreciate your review of our article and the valuable suggestions and comments you provided. Your input plays a pivotal role in enhancing the quality of the paper.

**1. Study rationale: What is the rationale to intervene to healthy elderly without hypertension, diabetes, etc? They are asymptomatic and doing well.**

**Our response:** Thank you for this thoughtful question. We selected healthy older adults as research subjects for the following reasons: 1) Even healthy older adults could experience reduced CRF based on our prior observations. In the second paragraph of the “Introduction” section (**Line 43-48, Page2-3**), we explained that age serves as an independent risk factor for the decline in CRF, not only diseases such as hypertension, diabetes, etc. 2) Healthy older adults with declined CRF may not exhibit obvious symptoms in a relatively quiet state. However, when engaging in essential daily activities such as climbing stairs, walking long distances for shopping, or participating in physical exercise, they may experience limitations, inevitably impacting their quality of life and independence. 3) Consequently, when addressing the issue of reduced CRF, healthy older adults are often overlooked compared to groups with underlying diseases. If moxibustion proves effective in enhancing the CRF of healthy older adults, it would bear significant importance in further enhancing their quality of life.

**2. Inclusion and exclusion criteria should be provided more clearly.**

**Our response:** We really appreciate your kind comment. It has come to our attention that our inclusion and exclusion criteria were somewhat unclear, with some content being repetitive.

Following careful deliberation, we have revised and redefined the inclusion and exclusion criteria. We added an inclusion criterion for healthy older adults, and deleted various disease conditions in the exclusion criteria to avoid duplication.

It is important to highlight that we have added an exclusion criterion according to the recommendation of another reviewer: “Participants with previous moxibustion treatment experience”. Given that real moxibustion treatment induces a noticeable warm sensation, unlike sham moxibustion, participants with prior moxibustion experience may discern the difference, potentially compromising the integrity of the study results. For specifics, please refer to **Line 107-121, Page 5-6**.

**3.** **Sample size calculation: this trial has 3 arms but sample calculation was done by 2 arms design. Adding additional 35 patients is missing reasonability.**

**Our response:** Thank you for bringing attention to this crucial issue. There does appear to be an inconsistency in calculating the sample size using this method. We have re-evaluated it using G-power software version 3.1.9.4. Sample size determination was based on one-way analysis of variance (ANOVA) comparing the VO_2peak_ value of three different groups. A Total sample of 107 participants is required to detect a significant difference with an effect size of 0.35 and 90% power at 5% level of significance (see picture below). Considering an estimated dropout rate of 15%, a total of 126 participants are needed, with 42 for each group. We have revised the “Sample size estimation section” (**Line 122-130, Page 6**) accordingly. Simultaneously, all other mentions of sample size in the manuscript have been adjusted, including the study flow chart (Fig 2).

**Reviewer #2:**

**This is a design paper for a study examining the improvement of exercise tolerance through moxibustion.**

**Our response:** Thank you for your careful review of this article. Your questions and comments are of great value to the improvement of our study protocol.

**The reviewer has some questions for this study.**

**1. Aren't people with experience in moxibustion excluded? If subject with experience in moxibustion is assigned to the Sham group, is there a possibility that the subject will realize that the sham procedure is being used?**

**Our response:** We really appreciate this great question! Given that moxibustion treatment induces a noticeable warm sensation, unlike sham moxibustion, subjects with prior moxibustion experience may discern the difference, potentially compromising the integrity of the study results. Therefore, as a precautionary measure, we propose **adding an exclusion criterion “With previous moxibustion history or experience”** to exclude participants with previous moxibustion experience. The pertinent modification is made in **Line 115, Page 6** of the manuscript.

**2. Isn't the effect of moxibustion different depending on the practitioner? Are differences between practitioners taken into consideration?**

**Our response:** Thank you for your insightful question. Although moxibustion is less operator-dependent compared to acupuncture, we recognize that variations may arise due to different operators. As far as we know, the factors influencing moxibustion's effectiveness primarily encompass different operators, type of moxibustion, chosen acupoints, moxibustion duration, frequency, and etc. In order to control the influence of these factors on the trial results,

**first,** we will minimize the number of practitioners and provide training to standardize their techniques, and homogenize the moxibustion operation process as much as possible, thereby mitigating the potential impact. We have an explanation regarding this under the “**Data collection, monitoring, and quality control**” section, please check **Line 326-330, Page 16** for details.

**second,** we meticulously control the other influencing factors, ensuring consistency across each group, including the utilization of the same moxa stick manufacturer, same acupoints, unified session duration and frequency, etc.

**3. What kind of consideration is given to placing moxibustion at the exact position of the acupuncture points?**

**Our response:** Thanks for your question. We believe this process involves two steps: accurately locating the acupuncture points and precisely positioning the moxibustion device on the acupoints.

**For first step,** standardized and precise methods for positioning acupoints are available. We will refer to *the 2006 National Standard of the People's Republic of China, "Acupoint Names and Locations" (GB/T12346-2006)*. The acupoint positioning details were elucidated in the “Moxibustion group” section (**Line 163-169, Page 8**) of the manuscript.

**For second step**, following accurate acupoints positioning, the practitioners use a marker to mark the acupoint locations on the local skin and subsequently secure the moxibustion equipment on the selected acupoints. Simultaneously, we utilize adhesive tape to firmly affix the moxibustion equipment, preventing any displacement during the moxibustion process.

In response to your query, we have made pertinent adjustments to the description of the moxibustion operation method to make it clearer. Please check **Line 173-193, Page 9** for specific details.

**4. What are the possible reasons for withdrawal from research? I think there is a possibility that moxibustion cannot be continued due to pain etc.**

**Our response:** We appreciate your insightful question. In clinical studies, it is essential to establish criteria for both dropout and withdrawal. For example, if a participant ceases treatment for personal reasons, it is categorized as dropout. Conversely, if the investigators request the subject's withdrawal from the study due to severe moxibustion-related side effects, it is classified as withdrawal.

We acknowledge that our previous explanations were not sufficiently clear. We only used "Dropout" as the heading, while the content also encompasses instances of withdrawal, such as severe side effects, inadequate treatment compliance, significant alterations in lifestyle habits, and weight changes exceeding 5 kilograms, etc**. (Line 224-231, Page 11).** Consequently, we have revised the heading to “Dropout and withdrawal", and listed different situations in different categories. Please refer to **Line 217-231, Page11** for specific details.

As for the participants you mentioned who withdraw from the study due to pain, we believe it is less likely as our moxibustion protocol is designed to impart a sensation of warmth to the subjects, not pain. Regardless, we appreciate your questions, as they led us to identify an issue in this section and correct the content.
